# Microbial Population Analysis of the Salivary Glands of Ticks; A Possible Strategy for the Surveillance of Bacterial Pathogens

**DOI:** 10.1371/journal.pone.0103961

**Published:** 2014-08-04

**Authors:** Yongjin Qiu, Ryo Nakao, Aiko Ohnuma, Fumihiko Kawamori, Chihiro Sugimoto

**Affiliations:** 1 Division of Collaboration and Education, Hokkaido University Research Center for Zoonosis Control, Sapporo, Japan; 2 Unit of Risk Analysis and Management, Hokkaido University Research Center for Zoonosis Control, Sapporo, Japan; 3 Administration Office, Hokkaido University Research Center for Zoonosis Control, Sapporo, Japan; 4 Department of Microbiology, Shizuoka Prefectural Institute of Public Health and Environmental Science, Shizuoka, Japan; Wageningen University and Research Centre, Netherlands

## Abstract

Ticks are one of the most important blood-sucking vectors for infectious microorganisms in humans and animals. When feeding they inject saliva, containing microbes, into the host to facilitate the uptake of blood. An understanding of the microbial populations within their salivary glands would provide a valuable insight when evaluating the vectorial capacity of ticks. Three tick species (*Ixodes ovatus*, *I. persulcatus* and *Haemaphysalis flava*) were collected in Shizuoka Prefecture of Japan between 2008 and 2011. Each tick was dissected and the salivary glands removed. Bacterial communities in each salivary gland were characterized by 16S amplicon pyrosequencing using a 454 GS-Junior Next Generation Sequencer. The Ribosomal Database Project (RDP) Classifier was used to classify sequence reads at the genus level. The composition of the microbial populations of each tick species were assessed by principal component analysis (PCA) using the Metagenomics RAST (MG-RAST) metagenomic analysis tool. *Rickettsia-*specific PCR was used for the characterization of rickettsial species. Almost full length of 16S rDNA was amplified in order to characterize unclassified bacterial sequences obtained in *I. persulcatus* female samples. The numbers of bacterial genera identified for the tick species were 71 (*I. ovatus*), 127 (*I. persulcatus*) and 59 (*H. flava*). Eighteen bacterial genera were commonly detected in all tick species. The predominant bacterial genus observed in all tick species was *Coxiella*. *Spiroplasma* was detected in *Ixodes*, and not in *H. flava*. PCA revealed that microbial populations in tick salivary glands were different between tick species, indicating that host specificities may play an important role in determining the microbial complement. Four female *I. persulcatus* samples contained a high abundance of several sequences belonging to Alphaproteobacteria symbionts. This study revealed the microbial populations within the salivary glands of three species of ticks, and the results will contribute to the knowledge and prediction of emerging tick-borne diseases.

## Introduction

Ticks (Acari: Ixodida) are globally one of the most important arthropod vectors of infectious diseases in animals and humans. They carry and transmit a range of bacterial, protozoan and viral pathogens, which are often zoonotic [1], [2], [3]. Opportunities for ticks to come into contact with humans and animals are increasing as their habitats change and their distribution widens. The incidence of tick-borne cases, including those emerging diseases, is on the rise [3], [4]. Recent studies have identified microorganisms, such as *Leptospira* and *Chlamydia*, which had previously not been associated with ticks [5], [6].

Ixodid ticks have four stages to their life cycle (egg, larva, nymph, and adult), and each post-embryotic phase requires blood to grow and molt. During feeding, ticks inject saliva into host animals to facilitate the uptake of blood. Tick saliva contains bioactive components that inhibit blood coagulation and the host immune system, and it also assists individuals to make a strong attachment to the skin of the host [7], [8]. Tick-borne pathogens, such as *Rickettsia*, *Ehrlichia* and *Anaplasma*, concentrate within the salivary glands, and are transferred into the host animal during feeding on blood [9], [10], [11], [12]. Electron microscopy has revealed the presence of non-pathogenic symbionts, such as *Coxiella*-like bacteria, in the salivary glands of ticks [13]. Symbionts have also been identified by molecular methods; however, the functions of these bacteria within tick salivary glands remain unclear [14], [15], [16], [17], [18]. Thus, the investigation of the microbial populations in the tick salivary glands may lead to a better understanding of the microbes that could be transmitted to the mammalian hosts together with tick saliva or that could play roles in tick biology such as by facilitating blood feeding.

High throughput sequencing has provided an insight to the diversity of microbes associated with ticks [5], [19], [20], [21]. The analysis of 16S ribosomal DNA (16S rDNA) amplicons by pyrosequencing is a high throughput technique that can be used to detect non-culturable microbes, and reveal entire populations of tick-borne microbes. The analysis of microbial populations found within tick salivary glands can provide important information to help predict emerging pathogens, assess potential risks, and understand the interactions between tick symbionts and pathogens.

In this study, tick samples were collected in the Shizuoka Prefecture, Japan, where Japanese spotted fever is endemic [22]. The aim of this work was to elucidate the microbial populations (including pathogens) associated with salivary glands of ticks using 16S rDNA amplicon pyrosequencing technology.

## Materials and Methods

### Sample collection and DNA preparation

Adult host-questing ticks of the species *Ixodes ovatus*, *I. persulcatus*, and *Haemaphysalis flava* were collected by flagging in mountainous areas of Shizuoka Prefecture from 2008 to 2010. No specific permissions were required for these locations and activities. Our field activities did not involve endangered or protected species. Table S1 indicates information about the sampling sites. The sample numbers of *I. ovatus*, *I. persulcatus*, and *H. flava* used for this study were 24 (14 female, 10 male), 12 (6 female, 6 male), and 5 (female only), respectively. Each tick was split into two parts (anterior and posterior) at the area between coxa 1 and coxa 2 using a microtome. The anterior part was then removed from the posterior part using sterile forceps. Since the salivary glands were attached with the anterior part, they could be removed from the tick carcass without damaging the midgut. The salivary glands were then collected into a sterile 1.5 ml tube using sterile forceps, followed by washing with sterile PBS in order to minimize bacterial contamination. All dissection steps were performed under a stereomicroscope with great care to avoid the contamination of the midgut fluid. Genomic DNA was individually extracted using QIAamp DNA Mini kit (QIAGEN, Tokyo, Japan) according to the manufacturer’s instructions, and stored at −20°C. Samples of *I. ovatus* female and male, *I. persulcatus* female and male, and *H. flava* female are indicated by IOf, IOm, IPf, IPm, and HFf, respectively, throughout (Table 1).

**Table 1:** Sequence results and number of detected genera.

Sample ID	Tick species	Sex	No. of sequence reads	No. of genera
IOf1	*I. ovatus*	female	5,664	10
IOf2	*I. ovatus*	female	4,484	10
IOf3	*I. ovatus*	female	3,498	6
IOf4	*I. ovatus*	female	4,712	7
IOf5	*I. ovatus*	female	5,591	3
IOf6	*I. ovatus*	female	4,200	5
IOf7	*I. ovatus*	female	5,030	6
IOf8	*I. ovatus*	female	5,634	23
IOf9	*I. ovatus*	female	7,643	8
IOf10	*I. ovatus*	female	5,636	7
IOf11	*I. ovatus*	female	4,049	2
IOf12	*I. ovatus*	female	7,275	14
IOf13	*I. ovatus*	female	3,351	3
IOf14	*I. ovatus*	female	7,048	7
IOm1	*I. ovatus*	male	4,986	13
IOm2	*I. ovatus*	male	3,790	12
IOm3	*I. ovatus*	male	7,916	22
IOm4	*I. ovatus*	male	3,844	5
IOm5	*I. ovatus*	male	6,340	18
IOm6	*I. ovatus*	male	7,130	22
IOm7	*I. ovatus*	male	6,176	22
IOm8	*I. ovatus*	male	9,788	28
IOm9	*I. ovatus*	male	9,628	12
IOm10	*I. ovatus*	male	7,170	17
IPf1	*I. persulcatus*	female	8,964	38
IPf2	*I. persulcatus*	female	3,599	16
IPf3	*I. persulcatus*	female	7,085	42
IPf4	*I. persulcatus*	female	8,242	40
IPf5	*I. persulcatus*	female	7,943	25
IPf6	*I. persulcatus*	female	10,506	18
IPm1	*I. persulcatus*	male	7,414	55
IPm2	*I. persulcatus*	male	8,173	19
IPm3	*I. persulcatus*	male	10,803	26
IPm4	*I. persulcatus*	male	10,144	34
IPm5	*I. persulcatus*	male	16,117	29
IPm6	*I. persulcatus*	male	9,221	40
HFf1	*H. flava*	female	6,438	3
HFf2	*H. flava*	female	8,339	44
HFf3	*H. flava*	female	6,204	5
HFf4	*H. flava*	female	10,017	18
HFf5	*H. flava*	female	8,294	24

### PCR amplification of V1 to V3 regions for 16S rDNA amplicon libraries

The V1, V2, and V3 hyper-variable regions of bacterial 16S rDNA were amplified by PCR using the universal primers 27F (5′-X-AGAGTTTGATCMTGGCTCAG-3′) and 518R (5′-ATTACCGCGGCTGCTGG-3′), corresponding to positions 27 to 518 of the *Escherichia coli* 16S rDNA [23], [24]. The 27F primer contained ten bases of a multiplex identifier (MID) tag sequence denoted as ‘X’. Primers 27F and 518R were modified with 5′-adapter A and 5′-adapter B sequences, respectively, for pyrosequencing (Roche, Basel, Switzerland). PCR was performed in a total volume of 50 µl, containing PCR Buffer, 0.2 µl of Platinum Taq DNA polymerase (Life technologies, Tokyo, Japan), 0.2 mM of each primer, 1 µl of 10 mM dNTP mixture, 1.5 µl of 50 mM MgCl_2_, and 1 µl of template DNA. The PCR reaction was preceded with a 2 min denaturation step at 94°C, followed by 30 cycles of 94°C for 30 sec, 55°C for 30 sec, and 72°C for 1 min. The quality of the PCR products (about 500 bp in length) were assessed by agarose (1%) gel electrophoresis, followed by purification using the Wizard SV Gel and PCR Clean-Up System (Promega, Tokyo, Japan). Concentration and quality of the amplicons were assessed with an Agilent 2100 BioAnalyzer (Agilent Technologies, Palo Alto, USA) using a DNA 1000 lab chip (Agilent Technologies).

### Pyrosequencing and data analysis

Amplicon libraries with different MID tags were mixed and subjected to pyrosequencing using a 454 Genome Sequencer Junior (GS-Junior; Roche) following the manufacturer’s protocol. The pyrosequencing data were deposited in DDBJ with accession no. DRA001731. The resulting data files (standard flowgram format, .sff files) were converted to FASTA files and sorted according to sample-specific MID tags using CLC Genomics Work Bench (CLC Bio, Tokyo, Japan). MID tag barcodes and primers were trimmed, then low-quality and short sequence reads (<150 bp) were removed. DECIPHER’s Find Chimeras web tool (http://decipher.cee.wisc.edu/FindChimeras.html) was used to remove chimeric sequences. The remaining reads were phylogenetically classified with the assistance of the Ribosomal Database Project (RDP) 16S Classifier version 10 (http://rdp.cme.msu.edu/index.jsp), which can accurately and rapidly provide assignments for domains to the genus level. A comparative analysis of each sample was performed using the MG-RAST metagenomics analysis server employing the RDP dataset (http://metagenomics.anl.gov/). Alpha diversity of each sample was also calculated using the MG-RAST server. Data sets were represented as the mean ± standard deviations (S.D.) after the Smirnov-Grubbs outlier test (α = 0.05).

### Conventional PCR methods

*Rickettsia*-specific PCR amplification of the citrate synthase gene (*gltA*) using the primers RpCS877p (5′-GGGGGCCTGCTCACGGCGG-3′) and RpCS1273r (5′-CATAACCAGTGTAAAGCTG-3′) [25] was performed on 22 samples that were highlighted by RPD analysis as containing the genus *Rickettsia*. PCR was performed in a total volume of 25 µl containing PCR Buffer for KOD-Plus-Neo, 0.5 µl of KOD-Plus-Neo DNA polymerase (Toyobo, Tokyo, Japan), 0.3 mM of each primer, 2.5 µl of 2 mM dNTP mixture, 1.5 µl of 25 mM MgSO_4_, and 1 µl of template DNA. The reaction started with a denaturation step at 94°C for 2 min, followed by 40 cycles of 94°C for 15 sec, 54°C for 30 sec, and 68°C for 30 sec, and a final extension step at 68°C for 2 min. PCR products were purified using ExoSap-IT (Affymetrix, Tokyo, Japan) according to the manufacturer’s instructions. Cycle sequencing was performed using BigDye v3.1 terminator chemistry (Applied Biosystems, Tokyo, Japan) and the same primers. Sequencing products were assessed using a 3130xl Genetic Analyzer (Life Technologies, Tokyo, Japan).

RDP analysis did not classify all sequences to the genus level. To characterize these sequences, the near full-length 16S rDNA gene was obtained from four *I. persulcatus* female samples by PCR using the universal primers fD1 (5′-AGAGTTTGATCCTGGCTCAG-3′) and Rp2 (5′-ACGGCTACCTTGTTACGACTT-3′) [26]. PCR was carried out in a final volume of 50 µl PCR Buffer for KOD-Plus-Neo, 1 µl of KOD-Plus-Neo DNA polymerase, 0.3 mM of each primer, 5 µl of 2 mM dNTP mixture, 3 µl of 25 mM MgSO_4_, and 2 µl of template DNA. PCR conditions started with a denaturation step at 94°C for 2 min, followed by 40 cycles of 94°C for 15 sec, 55°C for 30 sec, and 68°C for 45 sec, and a final extension step at 68°C for 2 min. Quality of the PCR products (approx. 1400 bp) was assessed by agarose (1%) gel electrophoresis, followed by purification of the products using the Wizard SV Gel and PCR Clean-Up System (Promega). PCR products were A-tailed and then cloned with TA cloning plasmids pGEM-T Easy (Promega). Ten clones per sample were randomly selected and sequenced.

### Sanger sequencing data analysis

Sanger sequencing data were analyzed using GENETYX version 9.1 (GENETYX Corporation, Tokyo, Japan). The GenBank accession numbers for the *gltA* sequences are AB911107 to AB911109, and the 16S rDNA sequences AB906824 to AB906829. Sequences were compared with those in public databases using nucleotide BLAST at NCBI website (http://blast.ncbi.nlm.nih.gov/Blast.cgi).

Phylogenetic analysis was conducted using MEGA version 6.05 [27]. The universal 16S rDNA sequences were aligned with those of closely related bacteria in GenBank using ClustalW and a maximum likelihood phylogram was constructed.

## Results

### Classification and quantification of bacterial taxa

**Figure 1:**
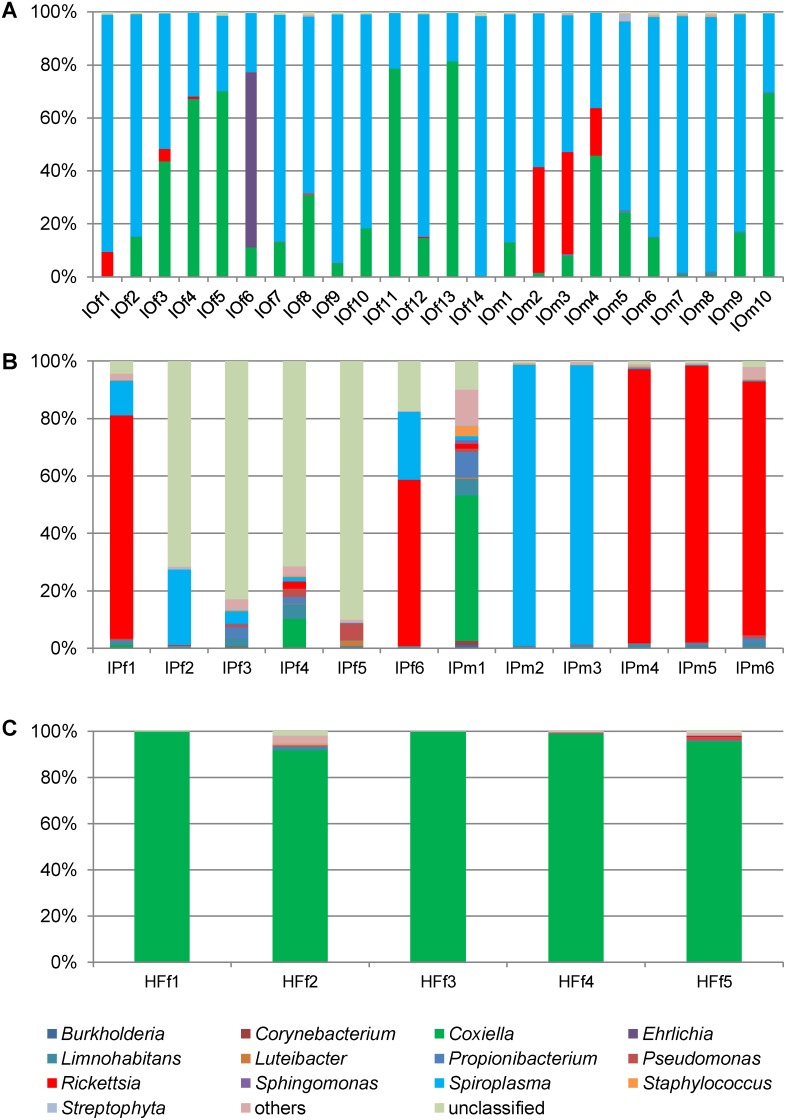
Relative abundances of different bacterial genera in the salivary glands of (A) *I. ovatus*, (B) *I. persulcatus* and (C) *H. flava*. All genera with less than 1.0% contribution were pooled into one group and labelled “others”.

Between 3,351 and 9,788 sequences were obtained for individual *I. ovatus*, of which almost 98% were assigned to the genus level (Table 1) (Figure 1A). A total of 71 bacterial genera were detected in *I. ovatus,* with 59 found in males and 37 in females. The two dominant bacterial genera were *Spiroplasma* and *Coxiella*, and these accounted for more than 90% of the bacterial community in ticks, except for a single *I. ovatus* female and 3 *I. ovatus* males (Figure 1A). *Rickettsia* (genus contains known tick-borne pathogens *R. japonica* and *R. helvetica*) was detected in ten samples, and *Ehrlichia* (genus contains known tick-borne pathogens *E. chaffeensis* and *E. muris*) was detected in two samples.

Between 3,599 and 16,117 sequences were recorded for individual *I. persulcatus*, with almost 82% assigned to the genus level (Table 1) (Figure 1B), except for those of four *I. persulcatus* females. Over 80% of the reads in these samples were unclassified at the genus level. At the phylum level, these reads were classified as Proteobacteria or Alphaproteobacteria by the RDP classifier (data not shown). There were 127 different bacterial genera detected in *I. persulcatus*, of which 92 were detected in males, and 81 in females. *Rickettsia* was detected in nine *I. persulcatus* (4 female, 5 male) individuals and *Ehrlichia* was detected in a single *I. persulcatus* male (IPm5).

Between 6,204 and 10,017 sequences were obtained for individual *H. flava*, of which almost 97% were identified to the genus level (Table 1) (Figure 1C). A total of 59 different bacterial genera were detected, and *Coxiella* accounted for more than 90% of the microbial population in all samples. *Spiroplasma* was not detected in any individuals of *H. flava*, despite appearing in all *Ixodes* samples. *Rickettsia* spp. were detected in three *H. flava* females, and no sample contained *Ehrlichia* spp. The details of microbial population analysis for each sample are presented in Table S2.

**Figure 2:**
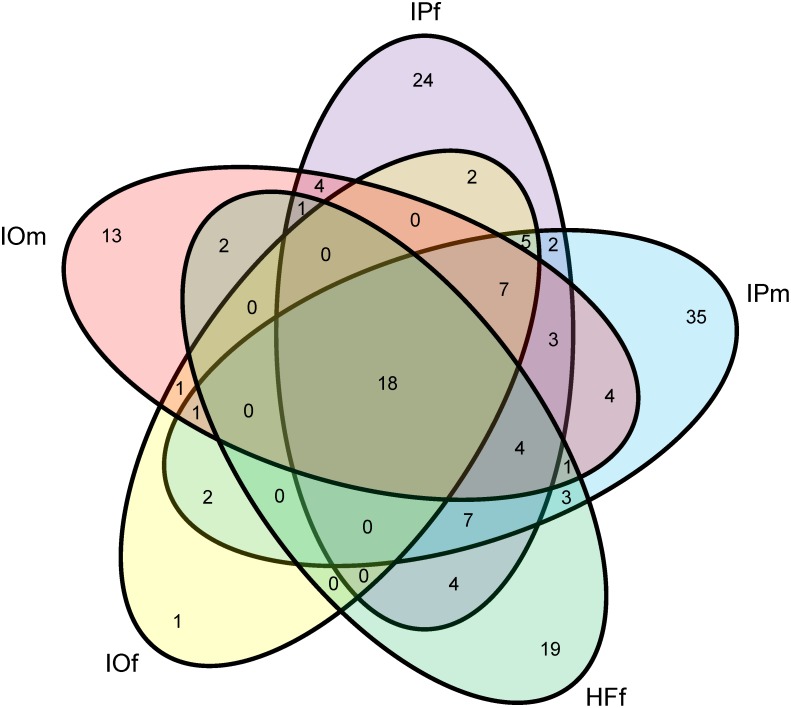
Venn diagram of all 163 identified genera distributed across the tick species and sex.

A summarized diagram of the number of bacterial genera detected in each tick group is presented in Figure 2. Out of 163 different genera identified, 18 were detected in all tick groups. These were *Acinetobacterium*, *Arcicella*, *Burkholdelia*, *Corynebacterium*, *Coxiella*, *Cryobacter*, *Curvibacterium*, *Flavobacterium*, *Limnohabitas, Methylobacterium*, *Novosphingomonas*, *Polynucleobacter*, *Propionilbacterium*, *Pseudomonas*, *Rickettsia*, *Sphingomonas*, *Staphylococcus*, and *Sterptophyta*. Some bacterial genera were uniquely associated with tick species or sex, i.e., IOf (1 genus), IOm (13 genera), IPf (24), IPm (35) and HFf (19).

### Comparison of microbiomes in salivary glands between tick species

**Figure 3:**
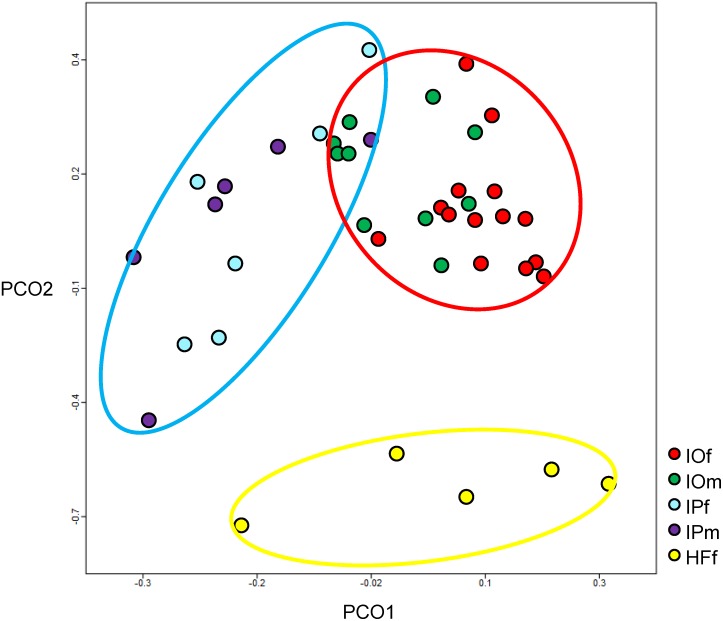
Principal component analysis of the bacterial composition in each tick sample. The plots were generated using the MG-RAST server. Each tick sample is shown in a different color depending on the species and sex of the tick; IOf, IOm, IPf, IPm, and HFf are respectively, shown in red, green, blue, purple, and yellow. The plots derived from the same tick species are highlighted in circles; *I. ovatus* (IO), *I. persulcatus* (IP), and *H. flava* (HF) are, respectively, highlighted in red, blue, and yellow circles.

PCA was performed using the MG-RAST server with normalized values and Bray-Curtis distance (Figure 3) for each tick sample. The microbial community composition of each sample clustered approximately according to tick species. The microbial populations of *Ixodes* and *Haemaphysalis* were completely separated by PCO2. The microbial community composition of *Haemaphysalis* ticks was broadly distributed along PCO1; however, in *I. ovatus* and *I. persulcatus* microbial populations were more distinct, but with some overlap within this component.

**Figure 4:**
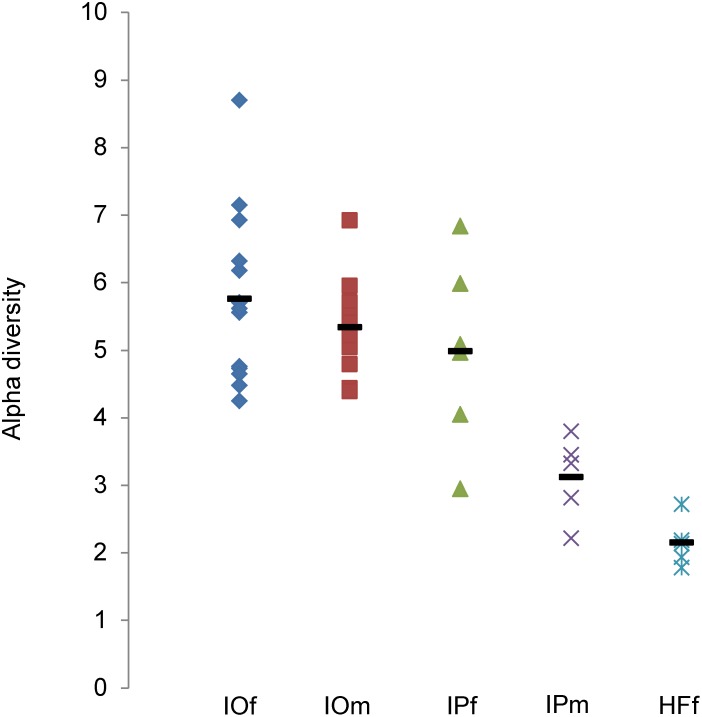
Alpha diversity calculated for each tick sample. The alpha diversity of each tick sample was calculated using the MG-RAST server. The mean value obtained for each tick group is represented by the horizontal line. Mean alpha diversity values: IOf (5.75), IOm (5.33), IPf (4.97), IPm (3.11), and HFf (2.14).

Alpha diversity for each sample was calculated using the MG-RAST server (Figure 4). Smirnov-Grubbs’s outlier test (α = 0.05) was used before the calculation of means and S.D. IPm1 was identified as an outlier and removed in the calculation for the mean value of IPm alpha diversity. Mean values were 5.75±1.19 (IOf), 5.33±0.72 (IOm), 4.97±1.25 (IPf), 3.11±0.55 (IPm) and 2.14±0.32 (HFf).

### Sequencing of *gltA*

**Figure 5:**
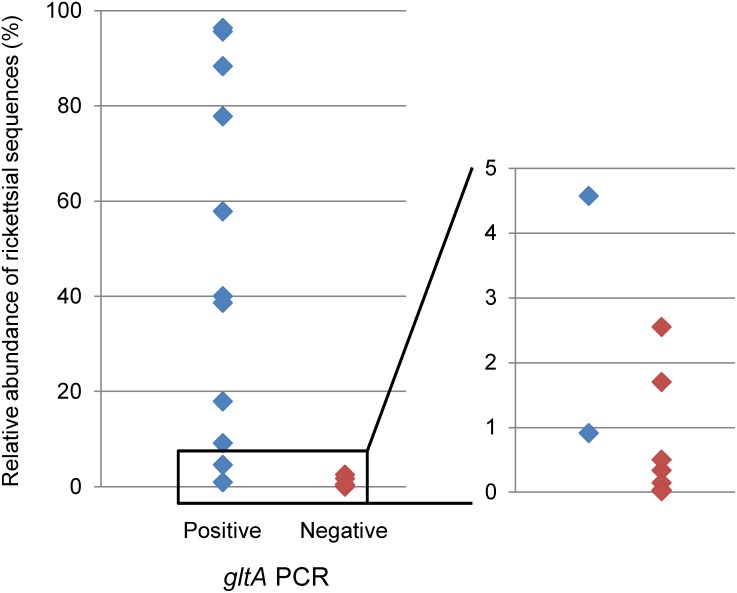
Comparison of the relative abundance of rickettsial sequences estimated by 16S amplicon analysis and the results of *gltA* PCR. Vertical axis represents the relative abundance of rickettsial sequences calculated from the data obtained from 16S amplicon analysis. Blue dots represent samples in which *Rickettsia* was detected by both 16S amplicon analysis and *gltA* PCR. Red dots represent samples in which *Rickettsia* was detected by 16S amplicon analysis but not by *gltA* PCR. The plots with relative abundance values between 0% and 5% are shown in the magnified graph provided in the right column.

The *gltA* gene was detected in 11 out of 22 samples previously identified as containing the genus *Rickettsia*. Samples that were *gltA*-positive tended to have a greater abundance of rickettsial bacteria than those that were negative (Figure 5). All *gltA*-positive samples were subjected to sequencing analysis. Each *gltA*-positive sample contained only one sequence type, indicating that individual ticks harbored bacteria carrying a single *gltA* allele. From 11 tick samples, three different *gltA* sequences were identified, and BLAST searches showed the highest identities (99.8% to 100%) with *R. asiatica*, *R. helvetica*, and uncultured *Rickettsia* sp. (Table 2).

**Table 2:** Summary of *gltA* sequencing.

SequenceID	Tick sampleID	Identity withreference (no.matched/no.nucleotides)	ReferenceGenBank no.	Rickettsiaspecies	GenBankno.
gltA_IOf1	IOf1	99.8% (438/439)	AB297808	*R. asiatica*	AB911107
gltA_IOf3	IOf3	99.8% (438/439)	AB297808	*R. asiatica*	AB911107
gltA_IOf4	IOf4	99.8% (438/439)	AB297808	*R. asiatica*	AB911107
gltA_IOm2	IOm2	99.8% (438/439)	AF394901	*R. asiatica*	AB911107
gltA_IOm3	IOm3	99.8% (438/439)	AF394901	*R. asiatica*	AB911107
gltA_IOm4	IOm4	99.8% (438/439)	AF394901	*R. asiatica*	AB911107
gltA_IPf1	IPf1	99.8% (438/439)	U59723	*R. helvetica*	AB911108
gltA_IPf6	IPf6	100% (394/394)	JN849396	Uncultured*Rickettsia* sp.	AB911109
gltA_IPm4	IPm4	99.8% (438/439)	U59723	*R. helvetica*	AB911108
gltA_IPm5	IPm5	99.8% (438/439)	U59723	*R. helvetica*	AB911108
gltA_IPm6	IPm6	99.8% (438/439)	U59723	*R. helvetica*	AB911108

### Sequencing of unclassified bacterial 16S rDNA

PCR fragments (1400 bp) were generated using universal primers to resolve the identities of sequences detected in four individuals of *I. persulcatus*. Between six and nine clones per sample were classified into Alphaproteobacteria (data not shown) based on BLASTn similarity searches. All the clones analyzed from two individual ticks were the same sequence type. There were four different sequence types in one individual, and two in another. These showed highest identities (99.5% to 99.7%) with uncultured Rickettsiales previously reported from *I. persulcatus* (GenBank accession number AF497583).

**Figure 6:**
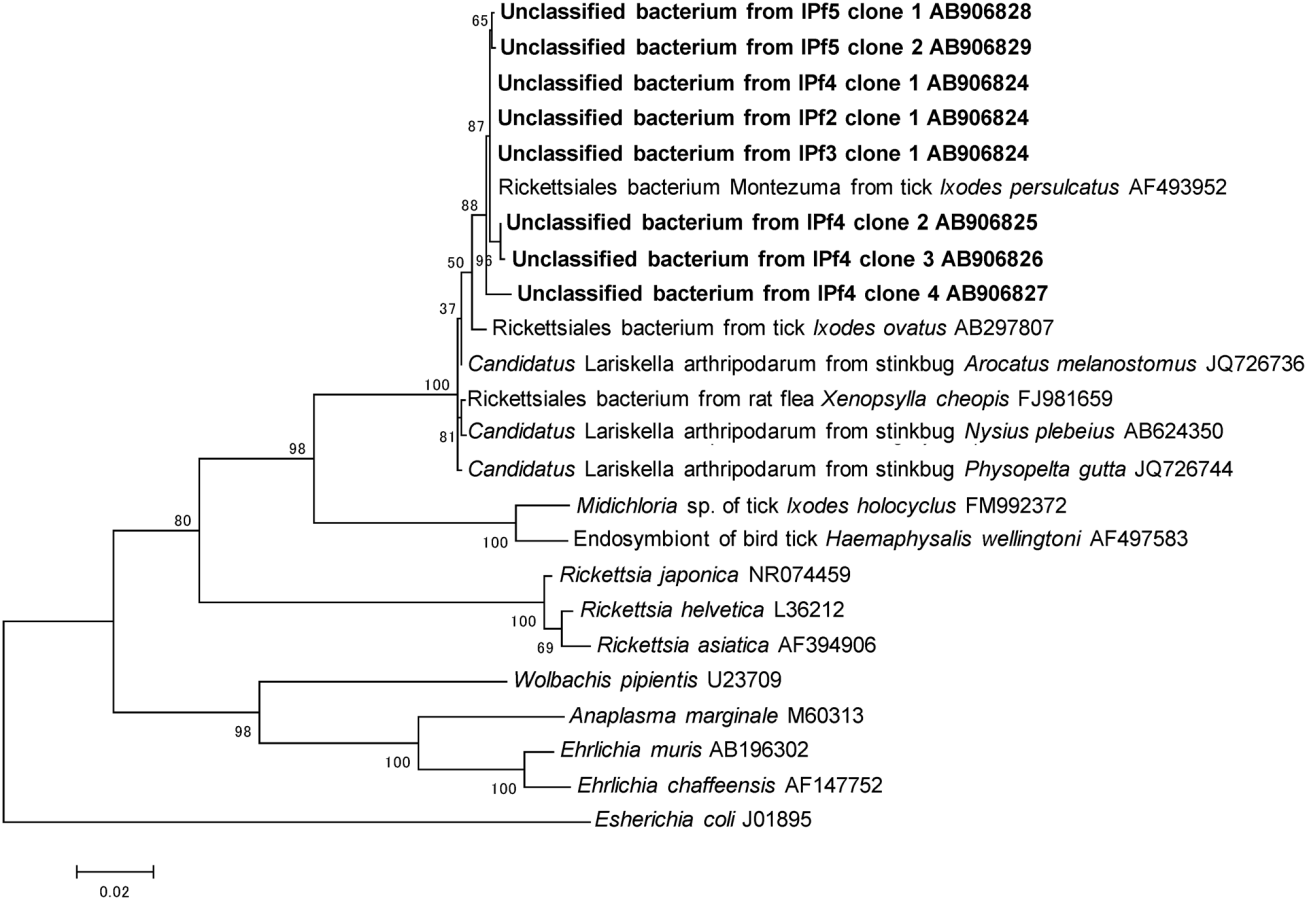
Phylogenetic analysis of the 16S rDNA sequences of unclassified bacteria from IPf2, IPf3, IPf4, and IPf5 using maximum likelihood method. The tree is rooted with the *Escherichia coli*. All bootstrap values from 1000 replications are shown on interior branch nodes.

Molecular phylogenetic analysis revealed that the Alphaproteobacteria from four *I. persulcatus* females clustered together within a single clade. This clade contains *Candidatus* Lariskella arthropodarum identified in several stinkbug species (*Arocatus melanostomus*, *Nysius plebeius*, and *Physopelta gutta*) and Rickettsiales derived from flea (*Xenopsylla cheopis*) and ticks (*I. ovatus* and *I. persulcatus*) [28], [29], [30], [31] (Figure 6).

## Discussion

The aim of this study was to assess and compare the diversity of bacterial populations within the salivary glands of *I. ovatus*, *I. persulcatus*, and *H. flava*. This is the first reported study of bacterial populations found in the salivary glands of different tick species. This metagenomic approach revealed bacterial populations totaling 163 different genera found in tick salivary glands. These included the genera of tick-borne pathogens such as *Ehrlichia* and *Rickettsia*. Further identification using species-specific PCR would be needed to clarify the presence of the tick-borne bacterial pathogens, such as *E. muris*, *E. chaffeensis*, *R. japonica* and *R. helvetica*, in the ticks used in this study [32], [33], [34], [35]. This combination of detection approaches may be useful for the screening and detection of possible pathogens in arthropod vectors.

*Rickettsia* was detected in 22 of the 41 (53.6%) samples by 16S rDNA amplicon pyrosequencing; however, only half of the 22 positive samples were positive with *gltA* PCR. This may be attributed to the relative amounts of rickettsial DNA in the PCR templates, where *gltA* PCR-positive samples tended to contain a higher proportion of rickettsial DNA than those that were negative (Figure 5). However, there were two *gltA*-negative samples (IPm1 and IPf4) that had higher proportions of rickettsial DNA than a *gltA*-positive sample (IOf4). This suggests that the sensitivity of conventional *gltA* PCR may be affected by the other factors such as the resolving power of agarose gel electrophoresis and the presence of PCR inhibitory components in samples [36], [37]. We suggest that a 16S rDNA amplicon pyrosequencing approach is a more sensitive method to detect specific pathogens.

Analysis of the *gltA* gene sequences from *I. ovatus* and *I. persulcatus* revealed that they belonged to *R. asiatica* and *R. helvetica*, respectively (Table 2). This result agrees with previous findings about the potential of the ticks to act as vectors for these rickettsia in Japan [38]. *R. helvetica* belongs to the spotted fever group of rickettsia and is a causative agent of febrile illness. A human case associated with this pathogens has been reported elsewhere [39], [40]. There was a high abundance (>70%) of this rickettsial species in some *I. persulcatus* samples (Figure 1B), suggesting that it is well adapted to the salivary glands of ticks, and waiting for transmission to mammalian hosts. In addition to pathogenic strains, the genus *Rickettsia* also contains symbionts associated with ticks. *Rickettsia*-like symbionts can influence the tick physiology, population dynamics, and the transmission of other pathogenic *Rickettsia* spp [41], [42].

*Coxiella burnetii* and *Coxiella*-like endosymbionts have been identified in several tick genera, including *Dermacentor, Ixodes, Haemaphysalis* and *Rhipicephalus* [43], [44], [45], [46]. *Coxiella*-like endosymbionts have been located at high densities in the salivary glands of the lone star tick (*Amblyomma americanum*) using fluorescence *in* *situ* hybridization [13]. The findings in this study also highlighted the presence of *Coxiella* in the salivary glands of three species of tick. The dominant presence of *Coxiella* in the salivary glands of ticks warrants further investigation to resolve their potential roles in tick biology, particularly blood-sucking behavior, and their interaction with other microbes.

The genus *Spiroplasma* contains a wide diversity of often unnamed or poorly characterized species, including non-pathogenic, symbiotic, and pathogenic organisms associated with a wide variety of arthropods. Symbiotic *Spiroplasma* has a close association with, and can affect the behavior of, their host arthropods. For example, Hurst *et al*. (2000) reported the preferential killing of males by *Spiroplasma*; when female insects (e.g., the butterfly *Danaus chrysippus*) are infected, the broods are female-biased because the infected male progeny die during embryogenesis [47]. One *Spiroplasma* sp. has been reported in ticks [48], [49], and it has also been associated with transmissible spongiform encephalopathy in humans and ruminants, although its role in the pathology of the host has not been clarified [50]. In this study, *Spiroplasma* was detected in *Ixodes* ticks, and not in *H. flava* (Figure 1). Previous research reported the genera *Spiroplasma* and the closely related *Mycoplasma* in several tick species in Japan [51]. The pathogenicity of *Spiroplasma* harbored in ticks in Japan is not known yet.

Results from the PCA of sequences indicated that microbial population structures in the salivary glands of ticks were different, and that samples from the same species of tick clustered together (Figure 3). Ticks can acquire microorganisms through a variety of ways, such as transovarial transmission, and from the environment, host animals during blood feeding, and mating partners. For microorganisms to exist in the salivary glands, they need to migrate from the midgut and enter the glands. The establishment of microorganisms within ticks can depend on the interactions between particular microbes, ticks and other symbioses [41], [52], [53]. The differences in the microbial populations within the salivary glands of tick species in this study were attributed to these complicated factors.

Previous studies revealed that tick microbial populations were different between developmental stages (egg, nymph, and adult) [19], [21]. The bacterial compositions also differed between organs, such as between midgut and ovary [19]. Some bacterial species, for instance *Borrelia burgdorferi* that is a causative agent of Lyme disease, exist in the midgut of the tick, moving into the salivary glands when stimulated by feeding on blood [54], [55]. For better understanding of microbial interactions with ticks as well as the potential pathogens transmitted by ticks, further study should include the comparison of the microbes between salivary glands and other organs. The analysis of the dynamics of microbial community composition during the process of feeding on blood may also uncover the roles of tick microbes.

The mean alpha diversity value (Figure 4) was greater for the female *I. ovatus* (5.61) than that of male (5.31). This rank order was also recorded for female (5.02) and male (3.38) *I. persulcatus* ticks, and may imply that some bacterial species preferentially select the gender of ticks. There may be some strategic biological relevance in the transmission of bacteria to mammalian hosts because female ticks feed for a longer period of time than males. The total number of bacterial genera (Table 1) detected in *I. persulcatus* (127) was greater than in *I. ovatus* (71).

Several *I. persulcatus* females contained unclassified bacteria belonging to the Proteobacteria and Alphaproteobacteria (Figure 1B). Based on the analysis of the nearly complete 16S rDNA sequences, the unclassified bacterial were classified into a single phylogenetic clade, which was recently proposed as a “*Candidatus* L. arthropodarum” clade [30]. This clade also includes Rickettsiales bacterium previously found in blood and biopsy samples of the patients with an acute fever disease, etiologically linked with tick bites [31]. The relationships between these microorganisms and their arthropod hosts are not clear, and their potential to act as causative agents of emerging tick-borne mammalian diseases warrants further investigation.

## Supporting Information

Table S1 **Longitude and latitude of sampling sites.**
(XLSX)

Table S2 **Details of classification results.**
(XLSX)
